# Serodiagnosis of sheeppox and goatpox using an indirect ELISA based on synthetic peptide targeting for the major antigen P32

**DOI:** 10.1186/1743-422X-7-245

**Published:** 2010-09-21

**Authors:** Hong Tian, Yan Chen, Jinyan Wu, Youjun Shang, Xiangtao Liu

**Affiliations:** 1 Key Laboratory of Animal Virology of Ministry of Agriculture, State Key Laboratory of Veterinary Etiologic Biology, Lanzhou Veterinary Research Institute, Chinese Academy of Agricultural Sciences, Lanzhou, Gansu730046, China

## Abstract

**Background:**

Sheeppoxvirus (SPPV), goatpoxvirus (GTPV) and lumpy skin disease virus (LSDV) of cattle belong to the *Capripoxvirus *genus of the *Poxviridae *family and can cause significant economic losses in countries where they are endemic. Despite the considerable threat that these viruses pose to livestock production and global trade in sheep, goats, cattle and their products, convenient and effective serodiagnostic tools are not readily available. Toward this goal, two synthetic peptides corresponding to the major antigen P32 were synthesized. These synthetic peptides were then used as antigen to develop an ELISA method to detect anti-SPPV and GTPV antibodies.

**Results:**

The results indicated that the optimal concentration of coated recombinant antigen was 0.2 μg per well for a serum dilution of 1:10. The ELISA performed favorably when sera from sheep immunized experimentally were tested.

**Conclusion:**

This assay offers the prospect of synthetic peptide as antigens for indirect ELISA to detect SPPV and GTPV antibody in sheep and goat sera.

## Background

Goat pox (GP) and sheep pox (SP) are malignant diseases of small ruminants causing heavy economic loss in the endemic countries. The diseases are endemic in India, Bangladesh, throughout the near and middle east, northern and central Africa [1,2]. The causative agents, sheep pox and goat pox viruses, belong to the genus *Capripoxvirus *in the family *Poxviridae *[3,4].

Although experienced veterinarians readily diagnose these diseases in their acute forms, low virulence strains and other exanthemas in sheep, e.g., orf or scabby mouth *Parapoxviridae *can present problems for differential diagnosis. Laboratory confirmation has been reliant upon classical virological techniques including animal transmission, electron microscopy for identification of virus in clinical material and virus isolation in cell culture [2]. Sero-epidemiological surveillance has been difficult since the only antibody tests available have been based upon immunofluorescence and virus neutralisation tests in cell culture. These tests are difficult and time consuming and not readily available in countries that do not hold live viruses.

P32, one of the structural proteins present in all the capripoxviruses, contains major immunogenic determinants. Here we selected two particular amino-acid sequence sits (residues 92-118 and 156-175, its character were identified by DNAstar Lasergene 7.1) and used this sequence for synthesis of one 27 amino-acid and one 20 amino-acid synthetic antigen. These synthesis peptides were used to develop a more convenient and cost-effective ELISA, that offers the prospect of reliable, high-throughput sero-surveillance on a flock or herd basis.

## Results

### Development of the indirect ELISA (I-ELISA) assay

By checkerboard ELISA, the optimal concentration of coating antigen was determined to be a total of 0.2 μg/well (including 0.1 μg A and 0.1 μg B). The optimal antibody dilutions were 1:10 for serum and 1:2000 for anti-goat HRP-IgG. Exposure time was optimally 45 min for serum samples and 15 min for conjugate at 37°C.

### Determination of the cut-off value

Receiver-operating characteristic curve analysis was used to set a cut-off value of 0.2, determined from a mean P/N of 1.516 with a SD of 0.056 for the 10 negative sera. The specificity and sensitivity of the assay at three different cut-off values, 0.1, 0.2 and 0.3, are shown in Table 1. At the cut-off value of 0.2, the sensitivity and specificity were relatively high. Thus, the cut-off values were established to be: positive ≥ 0.3; suspicious, 0.2-0.3; negative ≤ 0.2. Serum samples classified as suspicious were re-tested, with the sample judged to be positive if the result was confirmed by the repeat test.

**Table 1:** The specificity and sensitivity according to three different cut-off values

Cut-off value	Specificity	Sensitivity (positive serum)
0.1.	(73.33%)22/30	(100%)20/20
0.2	(90%)27/30	(100%)20/20
0.3	(100%)30/30	(95%)19/20

### Validation of the I-ELISA

The inter-assay CV ranged from 1.9% to 5.2%, and the intra-assay CV ranged from 1.5% to 5.0%, for 10 sera selected for validation testing (Table 2).

**Table 2:** The intra- and inter-assay coefficient of variation (CV) obtained from assessment of 20 sera

**Sample No**.	Inter CV(%)	intra CV(%)	**Sample No**.	Inter CV(%)	intra CV(%)
1	2.0	2.2	2	3.3	3.0
3	1.9	1.5	4	4.9	4.3
5	3.1	3.6	6	5.2	5.0
7	2.6	2.2	8	1.7	1.9
9	4.5	4.9	10	5.1	4.9

When detecting the immunity serum with I-ELISA, three sheep were positive for P32 antibody at 7 days post immunization, seven sheep were positive at 14 days post immunization, thirteen sheep were positive at 21 days post immunization, and all sheep were positive at 28 days post immunization (As shown in Table 3), and this results were confirmed by MNT.

**Table 3:** The result of detecting immunity antibody

Total immunity animals	I-ELISA									
	
	**0 day p.i**.		**7 days p.i**.		**14 days p.i**.		**21 days p.i**.		**28 days p.i**.	
	
	-ve	+ve	-ve	+ve	-ve	+ve	-ve	+ve	-ve	+ve
16	16	0	13	3	9	7	3	13	0	16

Note: "-ve" denoted negative; "+ve" denoted positive.

## Discussion

The P32 antigen is a structural protein present in all capripoxvirus isolates and contains a major antigenic determinant [5]. Thus, P32 is important in pathogenicity, diagnosis, prevention and control of capripoxvirus. Antibody detection enzyme-linked immunosorbent assays (ELISAs), based on mature virion envelope protein P32 expressed in *Escherichia coli (E. coli)*, have been developed previously for cattle [6] and sheep [7], but difficulties with expression and stability of the recombinant antigen have compromised these tests. Babiuk et al. (2009b) have recently developed an indirect ELISA, which uses inactivated, sucrose gradient-purified SPPV as coating antigen, for detection of antibodies to SPPV, GTPV and LSDV. However, although suited to screening sera from all three host species, the viral antigen is difficult and expensive to produce in large quantities.

Therefore, establishing ELISA detection methods with P32 protein as the antigen is essential, since in capripoxvirus-infected/immunity animals, late stage antibodies are mainly produced against P32 protein. So A and B peptide based on P32 main antigen region were synthesized, and incorporated A and B to develop an ELISA method for detecting SPPV and GTPV antibody in animals. Preliminary optimization of the assay showed that the best results were coated 0.1 μg each of peptide per well, followed by blocking with 10 mg/ml gelatin. These conditions allowed maximum OD_450 _absorbance. A crucial factor for establishing an indirect ELISA is eliminating potential false-negatives and false-positives that result from the variable antibody titres of different sera [8]. To address this issue, we performed ELISA on serial dilutions to determine an optimal cut-off using a ROC curve based on 30 P/N values. The obtained cut-off value gave an assay with a high degree of specificity and sensitivity. The assay also had good repeatability and promises to be useful in clinical contexts.

In summary, the I-ELISA established here was sensitive and specific for SPPV and GTPV antibody detection, and was easier to produce and perform, and less expensive, than existing serological methods for SPPV and GTPV antibody detection. The results suggest that the I-ELISA could be used to develop a reliable tool for large scale detection of SPPV and GTPV antibodies in herd tests.

## Conclusions

In this study we established an indirect ELISA using synthetic peptide target on P32 protein of capripoxvirus as antigen. The assay provides an alternative, inexpensive and rapid serological detection method that would be suitable for SPPV and GTPV antibody detection on a large scale.

## Materials and methods

### Serum samples

20 positive sera (isolated from naturally infected sheep and goats) were provided by the State Key Laboratory of Veterinary Etiological Biology Lanzhou Veterinary Research Institute. 10 negative sera were collected from SP-free sheep and goats herds in the Jingning region of GanSu. A total of 16 experimentally generated sheep sera were prepared by this laboratory.

### Peptides synthesis

The 27 amino-acid peptide (A) and 20 amino-acid peptide (B) were synthesized by Liberty (CEM, USA). Their experimental molecular weight, determined by mass spectrometry, were 3067.5 (theoretical: 3067.3) and 2475.8 (theoretical: 2475.7), respectively, and its purity, determined by HPLC analysis, was over 96%. Peptide solubility was 2.0 mg/mL in distilled water. The detailed sequences of the synthesized peptides are shown in Table 4.

**Table 4:** The sequence of synthesis peptide

Name	Amino-acid sequence	site	Length
A	EAKSSIAKHFSLWKSYADADIKNSENK	92-118	27
B	FHNSNSRILFNQENNNFMYS	156-175	20

### Development of indirect ELISA

A checkerboard titration was performed to determine the optimal working dilution of the coating antigen, serum and horseradish peroxidase-labelled rabbit-anti-goat IgG (HRP-IgG) (Sigma) using a 96-well ELISA plate. Antigen coating concentrations were 0.8 μg, 0.4 μg, 0.2 μg, 0.1 μg and 0.05 μg per well, and serum dilutions were 1:5, 1:10, 1:20 and 1:40. The dilutions that gave the maximum difference between positive and negative serum (P/N) by absorbance at 450 nm were selected for large-scale testing of serum samples. Test sera included positive, negative and blank sample controls. The reaction temperature, time and other conditions were optimized by P/N value [9].

After optimization, indirect ELISA was performed using the following procedure. Ninety-six well ELISA plates (Costar) were coated with 0.2 μg total synthetic peptides (0.1 μg A and 0.1 μg B) per well, diluted in carbonate buffer, and incubated overnight at 4°C. After three washes with phosphate buffered saline (PBS) containing 0.05% Tween 20 (PBST), plates were sealed with 10 mg/ml gelatin (Sigma), and incubated for 45 min at 37°C. After three washes with PBST, serum samples were diluted 1:10 in dilution buffer (PBS containing 5% skimmed milk, 10% horse serum), in a 100 μl volume per well, and incubated for 45 min at 37°C. After three washes, horseradish peroxidase-conjugated rabbit anti-goat serum (Sigma) was added in the same dilution buffer at an appropriate working concentration, 100 μl per well, and incubated at 37°C for 30 min. After three washes, color was developed with 3,3',5,5'-tetramethylbenzidine (TMB, Sigma), and the reaction was stopped after 15 min with 2.0 M H_2_SO_4_. The OD_450 _was read with a microplate reader (Model 680.Bio-Rad).

### Micro-neutralisation test

Capripoxvirus neutralizing antibodies were measured in sheep sera using Micro-neutralisation test (MNT) [10,2]. A total of 30 sera samples were tested for antibodies to SPPV and GTPV by the MNT. Positive serum had four replicates with every dilution. The negative serum, virus control and cell control steps were performed in quadruplicate. In this test, a positive serum titre was ≥1:32, negative serum and virus control had a CPE and cell control had no CPE or the test was repeated. Samples were considered positive or negative when the two well cells, mixed with serum at a 1:4 dilution, either exhibited or did not exhibit CPE. When only one well cells demonstrated CPE, the result was considered equivocal. This assay found 20 sera positive and 10 sera negative.

### Determination of cut-off values

To set negative/positive cutoff values, 20 positive and 10 negative samples were tested in duplicate by ELISA. The P/N value (OD_450 _of test serum/negative serum) of the 30 serum samples was compared with the MNT results. The end-point cut-off was determined by analysis of a receiver operating characteristic (ROC) curve based on 30 P/N values. Cut-off values were determined as the mean + 2 standard deviations (SDs) and mean + 3 SDs derived from the P/N values from the 10 negative samples. Estimates of diagnostic sensitivity and specificity were calculated using the three cut-off values.

For the test system to be valid, we determined that the OD_pos _should be higher than 0.5 and at least three times higher than the OD_neg_, so the OD_neg _should be lower than 0.2. For values outside these limits, the test was repeated.

### Validation, repeatability and comparison of the I-ELISA

To validate the test and evaluate repeatability, the co-efficient of variation (CV) was calculated between plates (inter-assay variation) and within the same plate (intra-assay variation) for 10 sera samples. For inter-assay CV, each sample was tested on four different plates on different occasions, and for intra-assay CV, four replicates within each plate were assayed. A total of 16 experimentally generated sera were evaluated by the ELISA as well as MNT.

## Competing interests

The authors declare that they have no competing interests.

## Authors' contributions

HT participated in peptides design, participated in the sequence alignment and drafted the manuscript. YC and JW carried out the MNT and established indirect ELISA. YS participated in the design of the study and performed the statistical analysis. XL conceived of the study, and participated in its design and coordination. All authors read and approved the final manuscript.

## Authors' details

Key Laboratory of Animal Virology of Ministry of Agriculture, State Key Laboratory of Veterinary Etiologic Biology, Lanzhou Veterinary Research Institute, Chinese Academy of Agricultural Sciences, Lanzhou, Gansu730046, China
